# In Fine Company

**Published:** 1881-06

**Authors:** 


*In Fine Company.*—Those who have been ambitious of the honor of having dentistry associated with medicine, and of having place and recognition by the medical fraternity as peers and equals, must have been gratified to read that at the session of the American Medical Association, which was held in Richmond lately, on motion of Prof. Gross, of Philadelphia, seconded by Drs. Sayre, of New York City, and Davis, of Chicago, a section of "Oral Surgery" (No. 7) was created, with Dr. D. N. Goodwillie, of New York, as Chairman and Dr. T. W. Brophy, of Chicago, Ill., as Secretary of the Section. Here is an opportunity for the ambitious to step forward and prove the claims of dentistry, as a "specialty of medicine," and doubtless "section No. 7" will be well worked up.

We are not sure that the dental profession need feel complimented at this tardy recognition. That it will prove of service to the profession we still more gravely doubt. Still, it is recognition, and this is what many have craved, and so far their ambitious cravings are satisfied. Will now the local medical societies admit the average dentist with his M. D. or without? Hardly. H.

